# Use of X-ray micro computed tomography imaging to analyze the morphology of wheat grain through its development

**DOI:** 10.1186/s13007-019-0468-y

**Published:** 2019-07-31

**Authors:** Thang Duong Quoc Le, Camille Alvarado, Christine Girousse, David Legland, Anne-Laure Chateigner-Boutin

**Affiliations:** 1 UR1268 BIA, INRA, 44300 Nantes, France; 2 UMR GDEC, INRA, Université Clermont-Auvergne, 63000 Clermont-Ferrand, France

**Keywords:** X-ray micro computed tomography, μCT, Image analysis, Wheat, *Triticum aestivum* (L.), Grain development

## Abstract

**Background:**

Wheat is one of the most important staple source in the world for human consumption, animal feed and industrial raw materials. To deal with the global and increasing population demand, enhancing crop yield by increasing the final weight of individual grain is considered as a feasible solution. Morphometric analysis of wheat grain plays an important role in tracking and understanding developmental processes by assessing potential impacts on grains properties, size and shape that are major determinants of final grain weight. X-ray micro computed tomography (μCT) is a very powerful non-invasive imaging tool that is able to acquire 3D images of an individual grain, enabling to assess the morphology of wheat grain and of its different compartments. Our objective is to quantify changes of morphology during growth stages of wheat grain from 3D μCT images.

**Methods:**

3D μCT images of wheat grains were acquired at various development stages ranging from 60 to 310 degree days after anthesis. We developed robust methods for the identification of outer and inner tissues within the grains, and the extraction of morphometric features using 3D μCT images. We also developed a specific workflow for the quantification of the shape of the grain crease.

**Results:**

The different compartments of the grain could be semi-automatically segmented. Variations of volumes of the compartments adequately describe the different stages of grain developments. The evolution of voids within wheat grain reflects lysis of outer tissues and growth of inner tissues. The crease shape could be quantified for each grain and averaged for each stage of development, helping us understand the genesis of the grain shape.

**Conclusion:**

This work shows that μCT acquisitions and image processing methodologies are powerful tools to extract morphometric parameters of developing wheat grain. The results of quantitative analysis revealed remarkable features of wheat grain growth. Further work will focus on building a computational model of wheat grain growth based on real 3D imaging data.

**Electronic supplementary material:**

The online version of this article (10.1186/s13007-019-0468-y) contains supplementary material, which is available to authorized users.

## Background

Wheat is one of the most important crops in the world with total annual production approximating to 750 million tons in 2016 [1], mainly used for human consumption, animal feed and industrial raw materials production (starch, gluten, ethanol). There is an increasing demand on wheat production due to the growing global human population. To overcome this increasing demand, one of the solutions is enhancing crop yield by increasing the final weight of individual grain [2–4]. There are many factors determining the final individual grain weight. Variations were reported between genotypes [5–7] and in response to changes in environmental factors such as temperature or water availability [5, 8]. Moreover, the final individual grain weight varies with its position along the spike and within spikelet [9, 10]. Several experimental studies have found an association between final grain weight and final grain size [6, 11, 12]. Wheat grain dimensions and shape are critical traits for wheat grain processing and milling; for example spherical grains with larger size are preferably selected for better performance of milling processes [13]. Moreover, the irregular shape of the wheat grain with a deep crease on one side makes the fractionation of the grain tissues problematical, resulting in various flour yield and properties [14]. Therefore, comprehensive knowledge of wheat grain growth, and of the determinism of its shape and size is of tremendous interest for plant science, global food supply and for several industries.

The final grain shape and size result from developmental processes occuring in various compartments of the grain. A wheat grain is composed of three major parts: the embryo, the starchy endosperm and the outer layers. Grain development is classically divided into three phases: early (lag) phase (from 10 to 15 days after anthesis), filling phase and maturation phase. After anthesis, the wheat grain mainly consists of the pericarp. The pericarp cells first divide and then extend by cell expansion [15]. In the early phase, the grain grows and leads to an ellipsoid shape and when cross-sectioned a heart-shaped grain with lobes separated by a crease. The endosperm and embryo develop in the embryo sac. The endosperm forms from free nuclei that divide first without being surrounded by cell walls (syncitium). Then cellularization takes place followed by cell division. At the end of early phase, the wheat grain reaches its final length [16, 17]. Then, the period of the filling phase takes place between 15 and 35–40 days after anthesis (the endosperm fills with storage compounds). The outer layers (pericarp, seed coat and nucellar epidermis) undergo drastic changes during this phase of grain development. The mesocarp or intermediate pericarp is made of parenchyma cells that undergo programmed cell lysis [15]. The endocarp or inner pericarp is a chlorenchyma tissue forming a cell layer in contact with the seed coat, this layer is photosynthetically active and stay active until late development while cells of the seed coat and nucellar epidermis collapse at the end of the development [15, 18]. At the end of the filling phase, the grain enters the maturation phase when it desiccates.

To better understand the role of the different tissues in grain growth, it is necessary to investigate the changes in the size and the shape of the tissues at different stages of development. Traditionally, investigations of the morphology/anatomy of plant tissues involves light microscopy with staining. More recently, confocal microscopy offers not only better image contrast with clearer discrimination between tissues types, but also the capability of 3D imaging. However, confocal imaging does not allow deep penetration into thick organs and requires the use of fluorescent probes or the presence of autofluorescent compounds in the tissues. Transmission electron microscopy is an alternative that enhances resolution. These are the prevalent 2D imaging techniques for obtaining morphological and biochemical information of cereal grains and in most cases only representative cross-sections taken in the middle of the grain are analysed, leaving the variability along the longitudinal axis unexplored [15, 19–23].

Plant growth and development is a 3D dynamic process. Its understanding requires to investigate changes in morphology not only in a middle slice of the grain, but within the whole grain. From the collection of 2D consecutive cross-sectional images, it is possible to generate complex 3D models of grains. For instance, morphological differences and tissue development were described in developing barley grain using 3D reconstruction from optical microscopy images [24]. 3D rendering using mass spectrometry imaging has also been applied for investigating biochemical information concerning distribution of biomolecules present in cereal grains [25, 26]. These studies used a common procedure for the 3D reconstruction of an individual grain: (1) sample preparation and sectioning, 2D image generation (2) stacking and alignment of images in ordered stacks; and (3) visualization of the 3D model of grain and analyses of microstructures in the grain. However, these imaging methods have considerable disadvantages. They require the steps of grain preparation sectioning and long acquisition time to cover the whole grain. For instance, Rousseau et al. [27] claimed that the acquisitions took 8 h for a single maize grain. Serial sectioning and 3D reconstruction to gain histological informations is therefore labour-intensive and time-consuming. It could cause the disruption of tissue structure and could not be applied to a large collection of samples [28]. This makes the quantification of dimensions and shape of the different tissues or compartments within the organ less reliable.

The limitations of 2D imaging techniques in investigating the 3D morphology and internal structure have led to the increasing use of 3D non-invasive imaging techniques such as X-ray micro-computed tomography (μCT) and magnetic resonance imaging (MRI), which enable investigations of 3D morphological characteristics of anatomical inner structures without the step of sectioning [29–33]. MRI and X-ray μCT do not provide the same information about the samples. MRI signal is based on water mobility while X-ray tomography principle is based here on the differential absorption of X-rays by the sample. In addition, the X-ray μCT can achieve much higher spatial resolutions than MRI [28, 32, 34]. As a result, the X-ray μCT has become more popular for gaining a comprehensive insights on 3D structures of plant tissues [35–37]. The contrast of images acquired by X-ray μCT theoritically depends on density, thickness and molecular structure of the sample [38]. In many studies, contrast agents have been used to enhance the contrast in X-ray μCT imaging [39, 40]. In the case of cereals, the resolution provided by μCT allows 3D imaging of the whole grain. Several studies were conducted on wheat grains to characterize the quality of the grains [30, 41], study sprouting and insect infestation [42], or compare the grain morphology between different genotypes [43] or different growth conditions [44]. Image analysis methodologies can quantify the 3D morphometry of grains such as grain dimensions (length, width and thickness) [27, 43, 44]. The proportion of tissues within the maize grain [27] or the crease depth of the wheat grain were also investigated [43]. However, these studies mainly focused on mature dry grains. In the case of developing grain, tissues are highly hydrated and may provide low contrast to X-rays. Therefore, the aim of this study was to investigate the validity of X-ray μCT without contrasting agents for better understanding the early development of wheat grain, and compare with the information that can be obtained from microscopy imaging. Moreover, we propose a semi-automated image analysis pipeline to quantify the morphology of wheat grain along its development. First, the 3D grain dimensions were quantified from 3D images. As different tissue types could be distinguished within the images, we developed a segmentation procedure to follow their evolution during development. Finally, we investigated the shape of the crease by computing average depth profiles that depict the growth of the lobes.

## Methods

### Plant materials

Wheat plants (*Triticum aestivum* L. cv. Recital) were grown as single plants in pots in a controlled green-house (day/night temperature: 20/15 °C day) at INRA Clermont-Ferrand (France) under conditions of natural day length. To harvest grains at different developmental stages, individual spikes were tagged at the beginning of anthesis and developmental stage of grains was calculated from this date on the basis of thermal time in Celsius degrees days after anthesis (°DAA) considering 0 °C as base temperature [45]. Eight stages of development were studied: 60, 80, 100, 120, 180, 240, 270 and 310 °DAA.

For each stage, 6 spikes were sampled and immediately transferred to the laboratory. One or two fresh grains per spike within the central spikelets on the spike, where the individual-grain weight is the highest [9, 10], were collected and selected for homogeneous fresh weight. Then, images of the whole grains were taken using a macroscope (MZ16F, Leica) in order to manually measure grain dimensions (length, width and thickness as shown in Fig. 11a).

### Light microscopy image acquisition

Microscopy cross-sections images of the equatorial region of wheat grain were acquired in order to visually compare the information obtained with microscopy and μCT.

Grain samples were fixed, embedded with London Resin White acrylic, cut and stained with toluidine blue O as described in [20, 21]. Bright-field images were acquired with a multizoom macroscope (AZ100M, Nikon).

### X-ray μCT image acquisition and reconstruction

The selected wheat grains were imaged using a commercial Phoenix Nanotom 180NF (GE sensing & Inspection Technologies GmbH, Wunstorf, Germany). The sample was inserted in a gelatin capsule with a droplet of paraffin to maintain it and avoid movement during scanning time; a small hydrated piece of paper was introduced at the top of the gelatin capsule in order to maintain relative humidity as high as possible in the capsule and then to limit grain desiccation during image acquisition. One fresh wheat grain was scanned per acquisition.

Various scanning settings were tested to adjust scanning parameters as beam-line energy and current, distance from detector to sample, number of 2D projection images and voxel size (resolution). These parameters were optimized to a satisfactory quality of image while keeping a short scanning time.

The wheat grains were scanned with a X-ray source energy of 30 kV and a current of 400 μA. During the scanning process, the sample was rotated and a projection image was recorded with a detector in 4 × 4 pixel binning mode, 12 image averaging and timing of 125 ms/image. A total of 600 2D projection images was generated. Output μCT scans were reconstructed with resolutions varying between 4.4 and 15 μm/pixel. Scanning time required between 1018 and 1740 s (depending on the developmental stage of the grain). The μCT image slices were stored in 16-bit TIFF format. The size of 3D images was between 550 and 950 voxels in the X and Y directions, and around 1100 voxels in the Z directions, resulting in typical file sizes around 1 GB. More detailed information about data are provided in Additional file 1.

### Experimental design for image processing and analysis

**Fig. 1:**
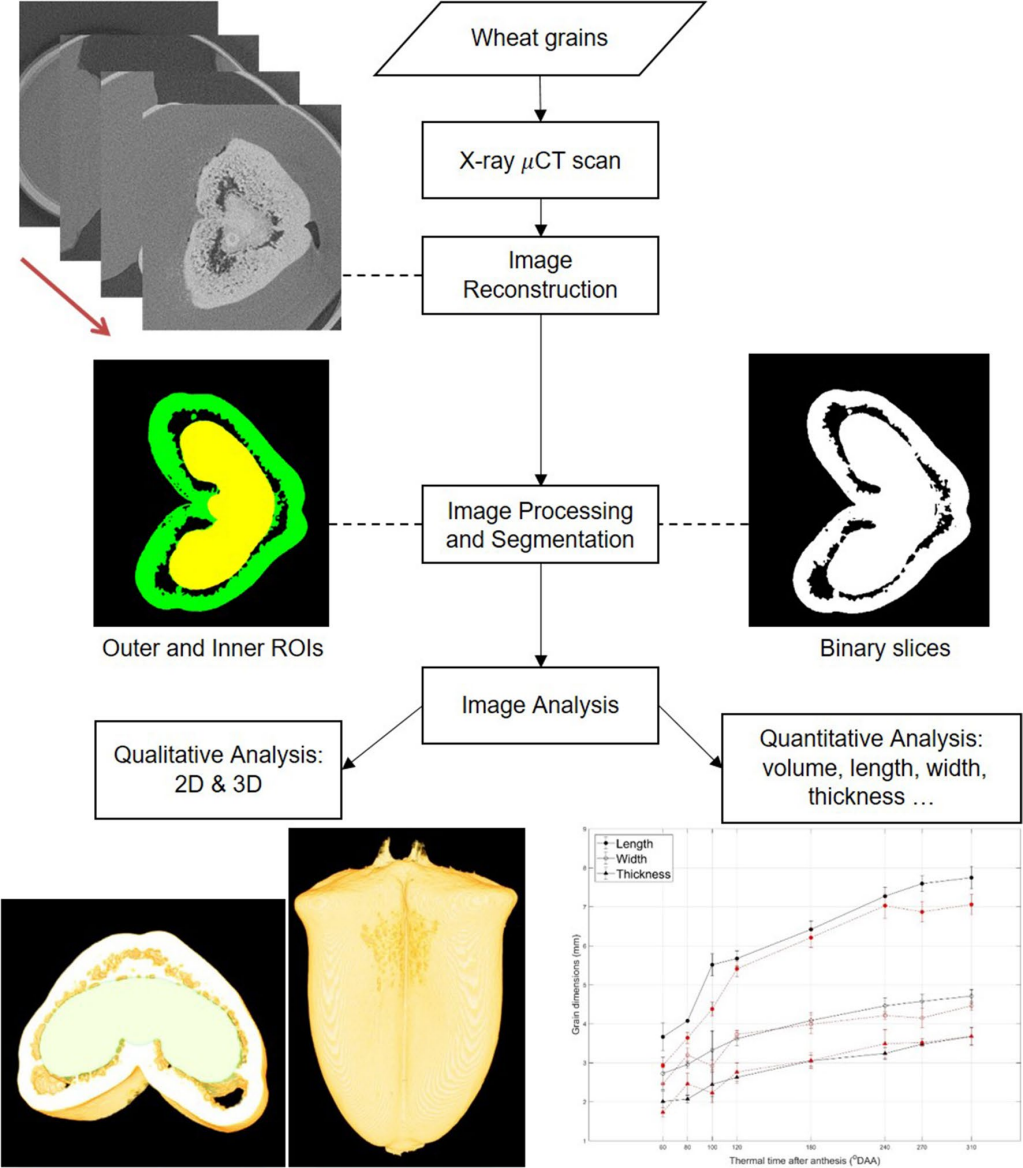
A flow chart of experimental design for μCT image acquisition and image analysis

A 3D volume of an individual grain is a stack of 2D images representing cross-sectional slices through the grain. From raw CT image slice data, stacks of binary images of all wheat grain samples were obtained by image processing and segmentation processes. These binary data were used to both quantitatively analyze the whole grain morphometry (volume, length, width and thickness) and provide a precise and accurate observation of overall structure, morphology and spatial distribution of the internal structures of the grain. The experimental design for μCT image acquisition and μCT image analysis is illustrated in Fig. 1. The workflow was implemented using MATLAB (The Mathworks, Natick, MA) and Fiji [46]. The full workflow together to a detailed description are provided in Additional file 2.

**Fig. 2:**
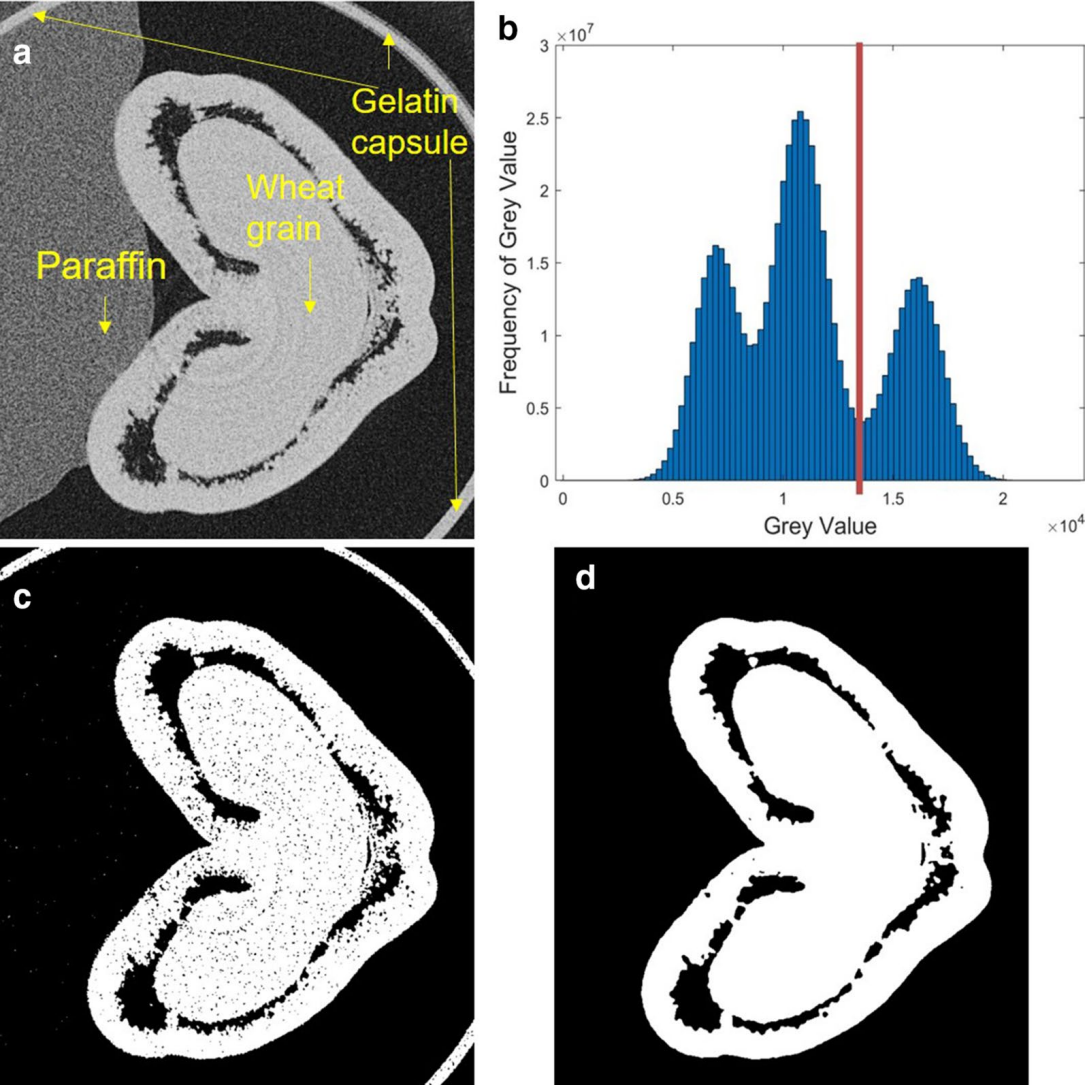
Workflow of wheat grain segmentation on MATLAB. **a** A μCT cross-sectional slice in 16-bit greyscale. **b** Cumulative histogram of all μCT slices of a sample. **c** Choice of global thresholding value using a histogram thresholding technique. **d** Region selection using 3D connected components, then median filter applied to smooth and remove noise from segmented image

### Wheat grain segmentation

All stacks of 2D cross-sectional slices, generated by μCT image acquisition setup contains voxel intensity information of wheat grain, paraffin and the gelatin capsule (Fig. 2a). Therefore, an image-processing pipeline, including global thresholding, region selection and morphological filtering was developed for the segmentation of the wheat grain within μCT image data.

We used a histogram thresholding technique for extraction of wheat grain information. This technique relied on selecting an appropriate global threshold on the histogram of the pixel intensity values that is able to filter the grain data from non-grain data and noise in each slice of μCT data. For each 3D image, a cumulative histogram of all μCT slices was generated. This histogram presents three peaks: the dark values corresponding to the background, the bright values correspond to wheat grain and the tube, and the intermediate values correspond to the paraffin (Fig. 2).

All pixels whose intensity value is greater than the local minima (on the left side of that third peak) were selected. This area on the cumulative histogram corresponds to the area of wheat grain and non-grain information, i.e. the gelatin capsule used for stabilizing the sample during acquisition time. This was done by computing the complement of that cumulative histogram and automatically identifying the second peak on the complemented histogram which is the suitable threshold value.

A three-dimensional connected components algorithm using the default connectivity of 26 was subsequently applied and the largest component was selected as the region of grain. A median filter was then applied to reduce noise on the segmented μCT slices. The radius of the median filter ranged from 2 to 3.

### Wheat outer and inner tissues segmentation

**Fig. 3:**
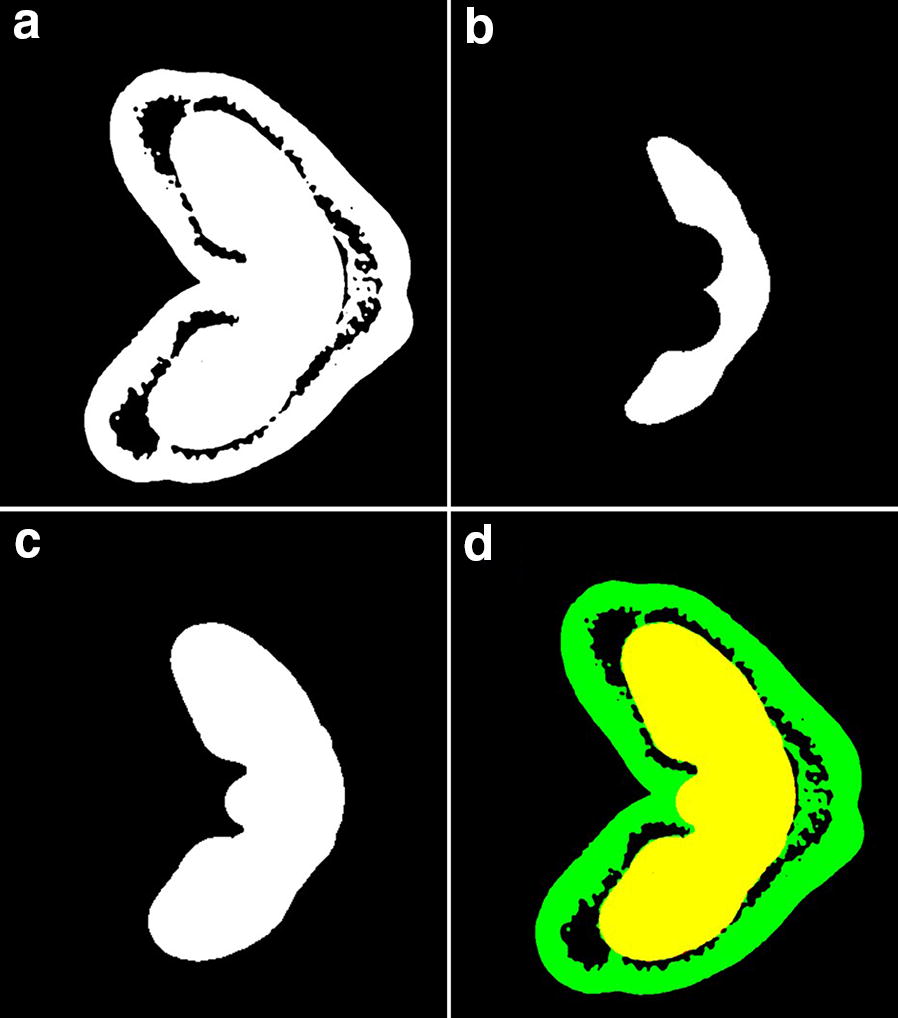
Workflow of wheat outer and inner tissues segmentation on FiJi. **a** A μCT cross-sectional slice in 8-bit binary; **b** Result of erosion on **a**; **c** Result of dilation on **b**; **d** Superimposition of **c** over **a**. The green region represents the wheat outer tissues and the the yellow region represents the wheat inner tissues

The segmentation step aims at partitioning the 3D images into three main regions: the outer and inner tissues, and the voids within the grain. On the μCT images, the similarity in contrast between outer and inner tissues causes difficulties on tissue differentiation based on voxel intensity. We thus considered that the region of inner tissues is compact, large and bright, and partly surrounded by voids. The region of inner tissues was identified by applying a morphological opening on 3D images of wheat grain (Fig. 3). Morphological opening is an operator from mathematical morphology that removes bright structures whose thickness is smaller than a structuring element of a given shape and size [47, 48]. Morphological opening is obtained by applying morphological erosion operation followed by a dilation operation, using the same structuring element for both operations. The morphological erosion computes the minimum values within structuring elements centered on current voxel. It has effect on (1) removing voxels belonging to the region of outer tissues, (2) reducing the size of the region of inner tissues. The dilation estimates the maximum value within structuring elements centered on current voxel. It results in restoring the initial shape of the region of inner tissues. A spherical structuring element was used in order to better preserve the shape of the biological structures. The radius of the sphere was chosen manually for each image depending on the image resolution and on the actual thickness of the outer tissues.

**Fig. 4:**
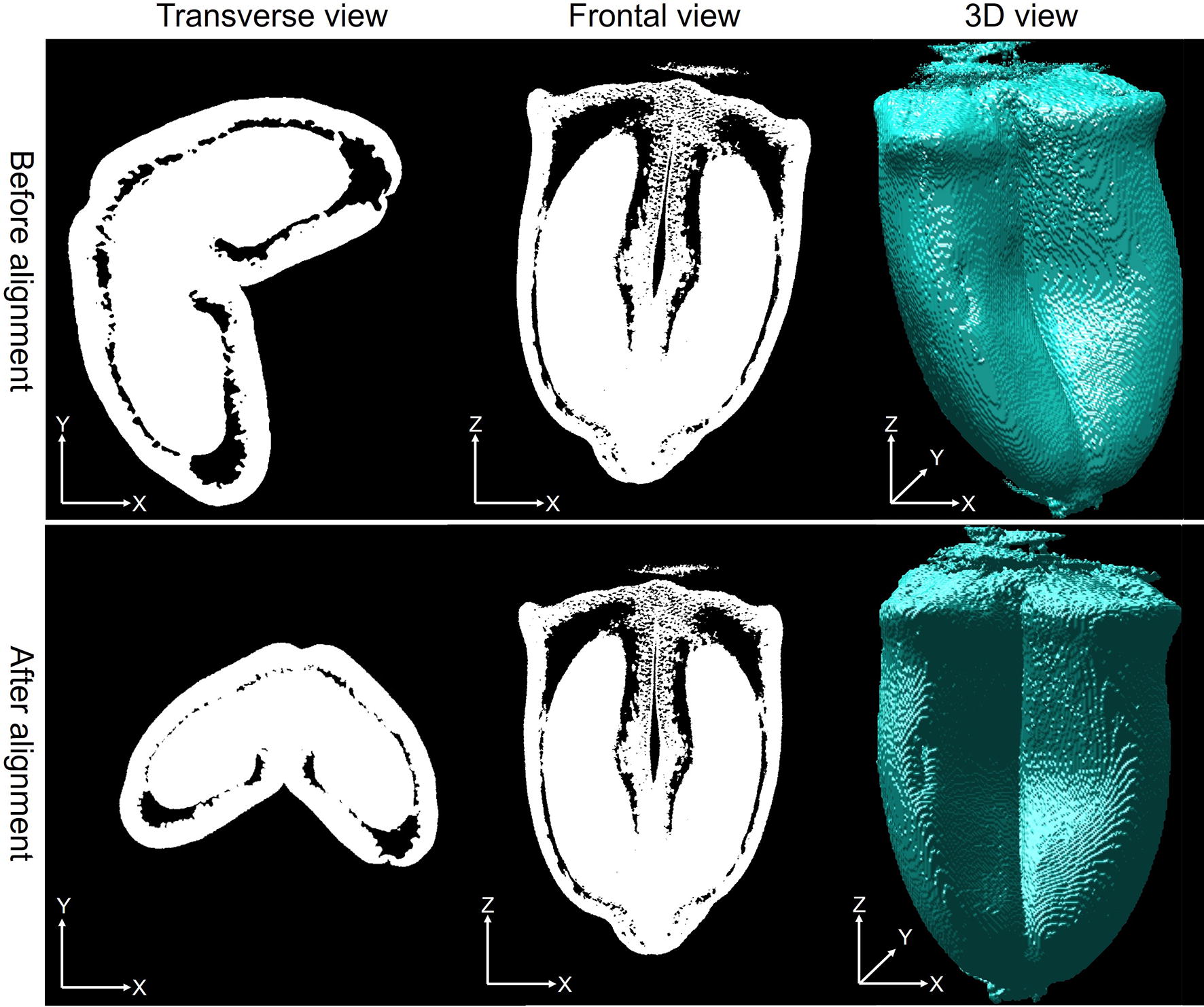
Different views of a 120-°DAA sample before and after alignment

The time needed for running the wheat outer and inner tissues segmentation increases with the cube of the radius of the structuring element. As large structuring elements were used, processing the whole data set could require several computation days. In order to accelerate the processing, reducing the size of the input data was considered. By downsampling by 2 the size of 3D images of wheat grain before performing the morphological closing, the computational time of a typical image sharply decreases from nearly one day to 2–3 h. The output of the closing operation is then upsampled by 2 to the original size of input 3D images (Fig. 3d).

### Alignment of μCT image data

3D images of wheat grains were acquired with various positions and orientations. It thus led to difficulties in not only making a direct comparison between individual grains based on cross-sections (Fig. 4), but also estimation of morphometric features of wheat grains. To facilitate this, the 3D images of binary grains were aligned into a predefined position to provide consistent orientation and position within the collection.

Assume that 3D images of the grain mathematically locates on a 3D Cartesian coordinate system where XY plane corresponds to the transverse view, ZY plane corresponds to the frontal view and XZ corresponds to the sagittal view. All grains were aligned by performing the following steps:

1. A 3D translation was applied to re-center the grain. The translation vector was obtained as the difference between the centroid of the grain and the center of the target image.
2. A rotation around the Z axis was performed to align the crease of the grain with the YZ plane (see the transverse view in Fig. 4). The rotation angle corresponded to the average angle of the ellipse with same normalized second moments computed on 200 slices around the middle slice.
3. A rotation around the Y axis was performed to align the main axis of the grain with the Z-axis (see the frontal view and 3D view in Fig. 4). Again, the rotation angle corresponded to the average angle of the equivalent ellipse computed on 200 XZ-slices around the middle slice in the Y direction.
4. Depending on each individual 3D image, a rotation around X or the previous steps have to be performed to obtain an appropriate alignment of the wheat grain data. In practice, all grains were re-sampled within a 3D image with $1000\times 1000\times 1200$ voxels.

### Morphometric features extraction

**Fig. 5:**
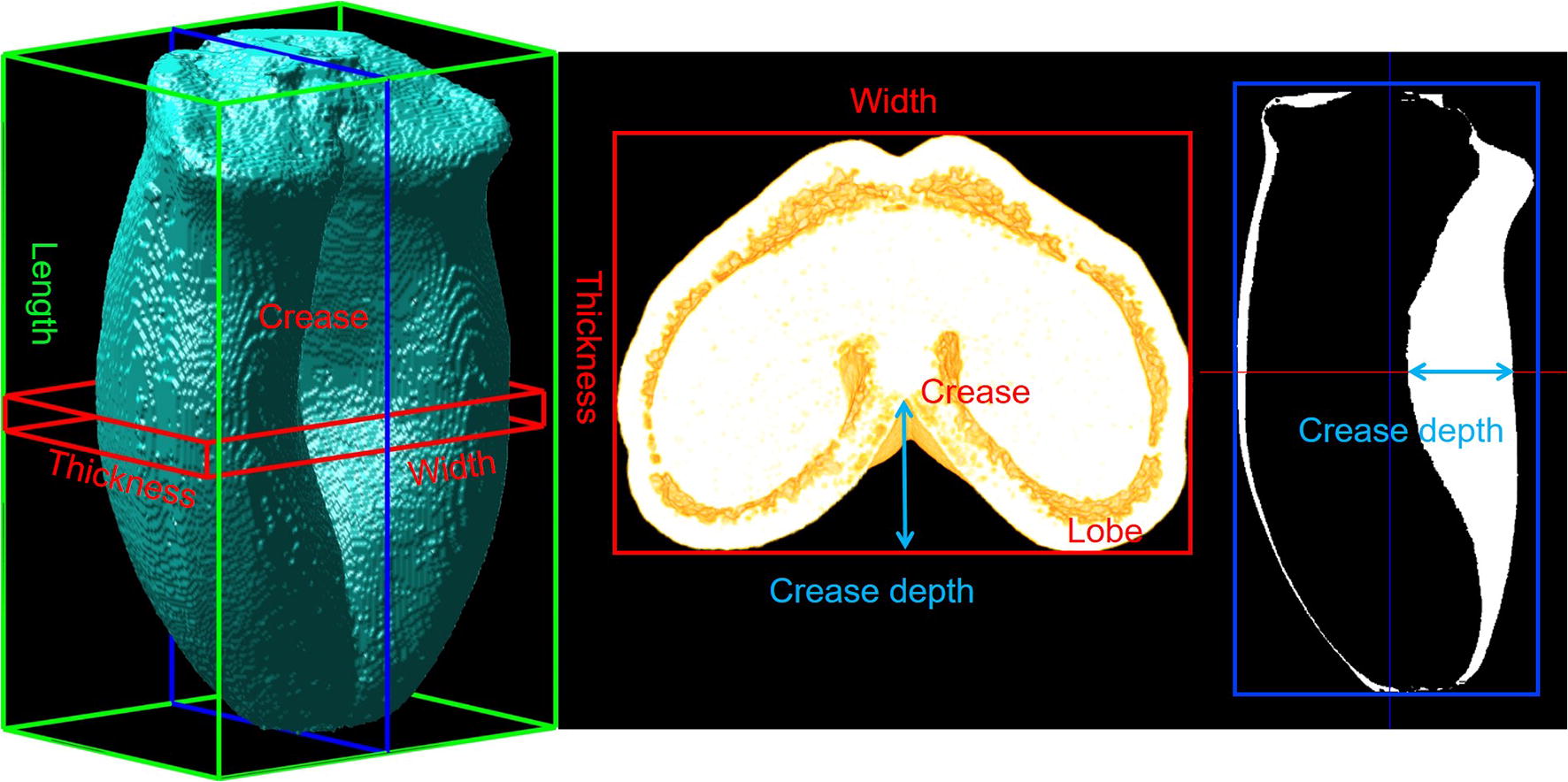
Morphometric measures of wheat grain

Grain length was computed as the length of the bounding box fitted on the aligned wheat grain. In order to estimate the width and thickness, 2D bounding boxes were fitted on the group of μCT cross-sections around the middle of grain. Then, the width and thickness were computed as the average dimensions of those 2D bounding boxes (Fig. 5).

The grain’s volume as well as the volume of wheat outer and inner compartments were measured as the total number of white pixels (corresponding to pixels with values of 1 in binary image) obtained by previous segmentation process.

The volume of voids inside the grain was measured as the total number of black pixels (corresponding to pixels with values of 0 in binary image) within regions determined by wheat grain.

By multiplying with the resolution (mm) of each stack of μCT slices, the morphometric measures of each individual grain were represented in mm and mm^3^ units.

### Crease depth

A heart shape with a deep crease is a distinctive feature of the grain of most cultivated wheat lines. It is not found in other cereal grains such as rice or maize. The measure of crease depth on the middle slice of mature grain was investigated by Strange et al. [43]. Distribution of crease depth values across all slices of segmented wheat grain is a new proposed morphometric feature which is investigated. This measure helps us understand how shape as well as the values of crease depth are spread over the length of wheat grain.

**Fig. 6:**
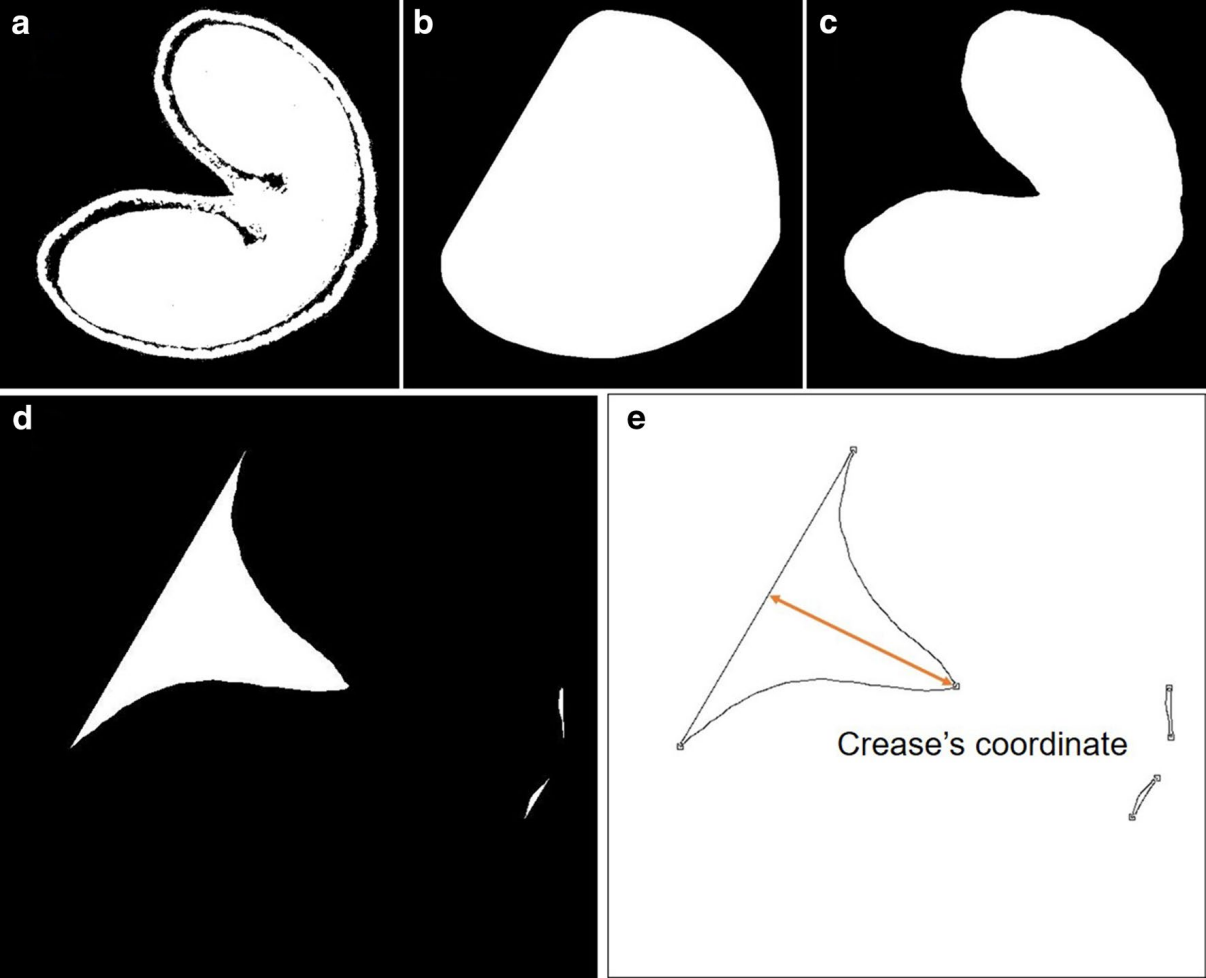
Workflow of crease detection on a μCT slice. **a** An input binary μCT slice, **b** convex hull of the input slice. **c** Filled image generated from input slice. **d** Difference between convex hull and filled image. **e** Crease’s coordinate was detected by using the CPDA corner detector, and crease’s depth was calculated by mathematical formulations

The crease depth was computed by detecting crease’s coordinate on each cross-sectional image of segmented wheat grain CT data. From each cross-section, an image of convex hull and a filled image were generated. The difference between the filled segmented image and the convex image allowed to identify the region that contains crease’s position (Fig. 6d). It could be detected by using the Chord-to-Point Distance Accumulation (CPDA) detector [49]. CPDA is a multi-scale corner detection technique that detects the corners of a region by estimating the curvature of its boundary curve. In practice, the boundary is extracted from edge image detected by Canny edge detector [50]. The three points corresponding to largest curvatures were selected. The highest point in the image corresponded to the position of crease. The distance between crease’s position and the opposite side region was used as the estimated crease’s depth (Fig. 6). The evolution of crease depth value along the cross-sections was used to depict the shape of the crease.

## Results

### X-ray μCT image acquisition and reconstruction

**Fig. 7:**
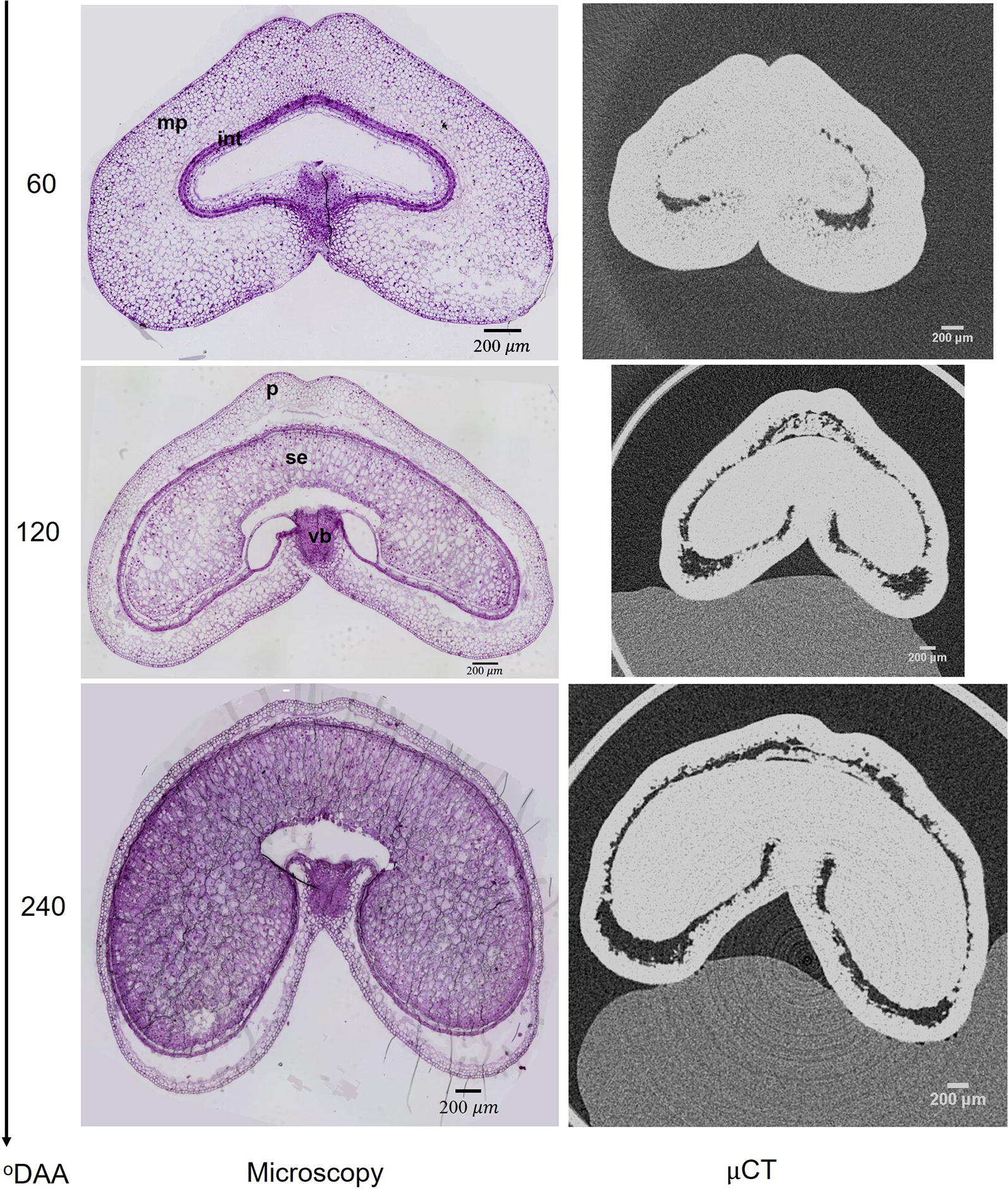
Cross-sections of wheat grain observed by light microscopy after staining with toluidine blue or obtained by X-ray μCT at 60, 120 and 240 °DAA. *int* integuments, *mp* mesocarp, *p* pericarp, *se* starchy endosperm, *vb* vascular bundle. Scale bar is 200 μm

X-ray μCT was carried out on fresh developing wheat grains at different stages from 60 to 310 °DAA and at a spatial resolution varying from 4.4 to 15 μm/pixel. These resolutions enabled the observation of the external shape of the grains as well as the distinction between an outer and an inner compartments separated by a dark region (Fig. 7).

**Fig. 8:**
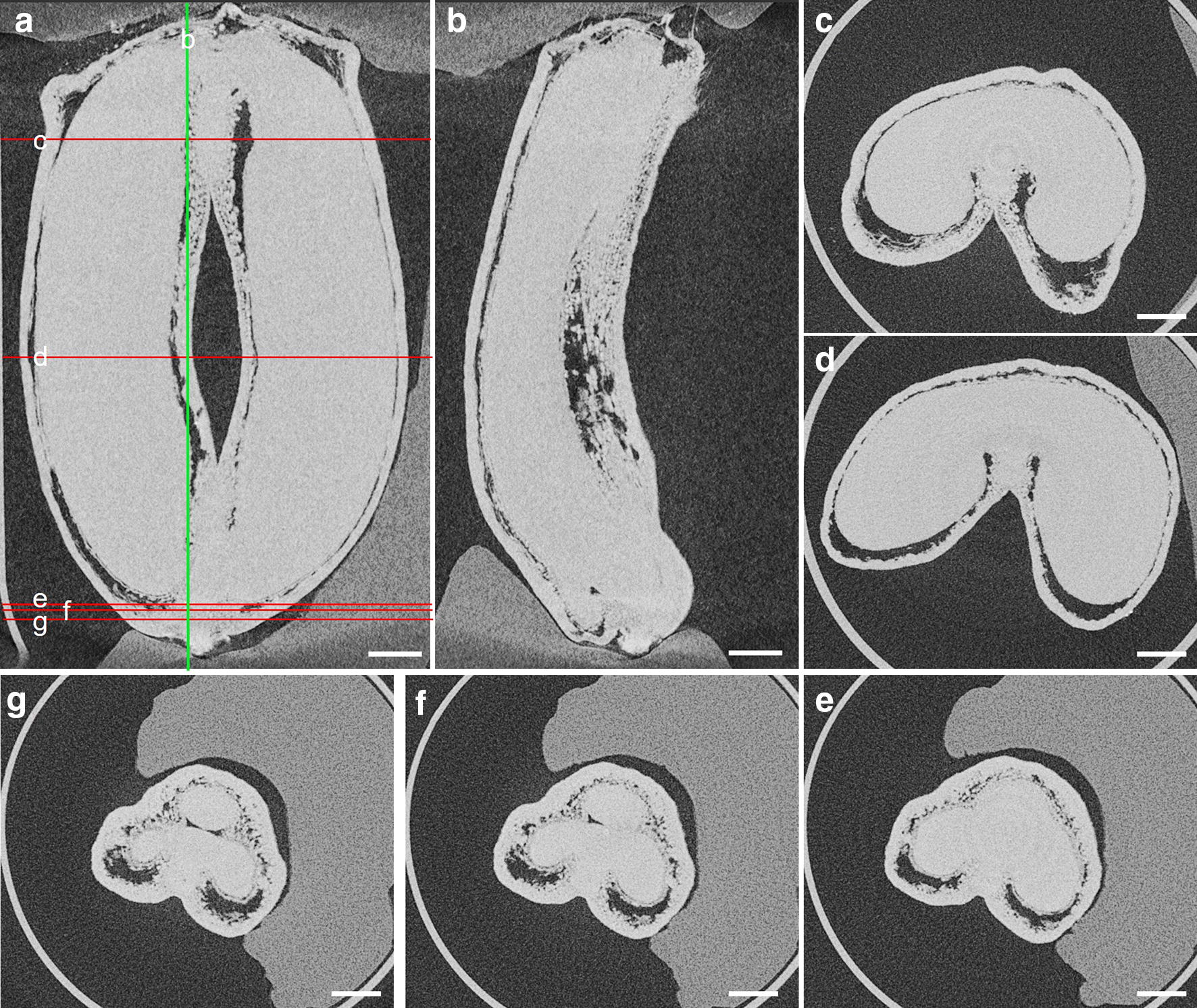
Cross-sections at different positions of a wheat grain sample at 240 °DAA. **a** A frontal slice of the grain; **b** A sagittal slice of the grain corresponds to the green line on **a**; **e, f, g** Cross-sections at the bottom of the grain, which correspond to red lines on **a**, reveal the embryo’s location. Scale bar is 600 μm

The outer compartment observed in the μCT images corresponds to the outer pericarp of the grain (epicarp and mesocarp) in the microscopy images. The inner compartment contains several tissues that evolve across development: the internal pericarp, the nucellar epidermis, the integuments at the earliest stages, and the seed coat and endosperm later from 80 °DAA. This latter tissue is visible on microscopy images only from 80 °DAA, since at 60 °DAA endosperm cells are not yet formed in the endosperm cavity. The dark regions highlighted in the μCT images are voids resulting from the lysis of the mesocarp tissue. In contrast, microscopy images shows empty spaces in the endosperm cavity that do not appears as dark region in μCT images. The endosperm cavity is dense to X-ray and therefore is filled. Several structures observed with light microscopy are not visible with X-ray μCT with our experimental settings. This is the case, for instance, for the vascular bundle located close to the crease.

Appropriate software such as FiJi allows for fast and easy virtual travel within the 3D images of the grain to visualize internal structures and highlight variability in shape, size and density. This is illustrated in Fig. 8 with variations in shape and size of the two lobes of the grain along the longitudinal axis. The embryo is revealed at the bottom of the grain from 180 °DAA. μCT allows the distinction between embryo and other internal compartments (Fig. 8g), while this separation becomes unclear above (Fig. 8e, f).

### 3D grain segmentation

**Fig. 9:**
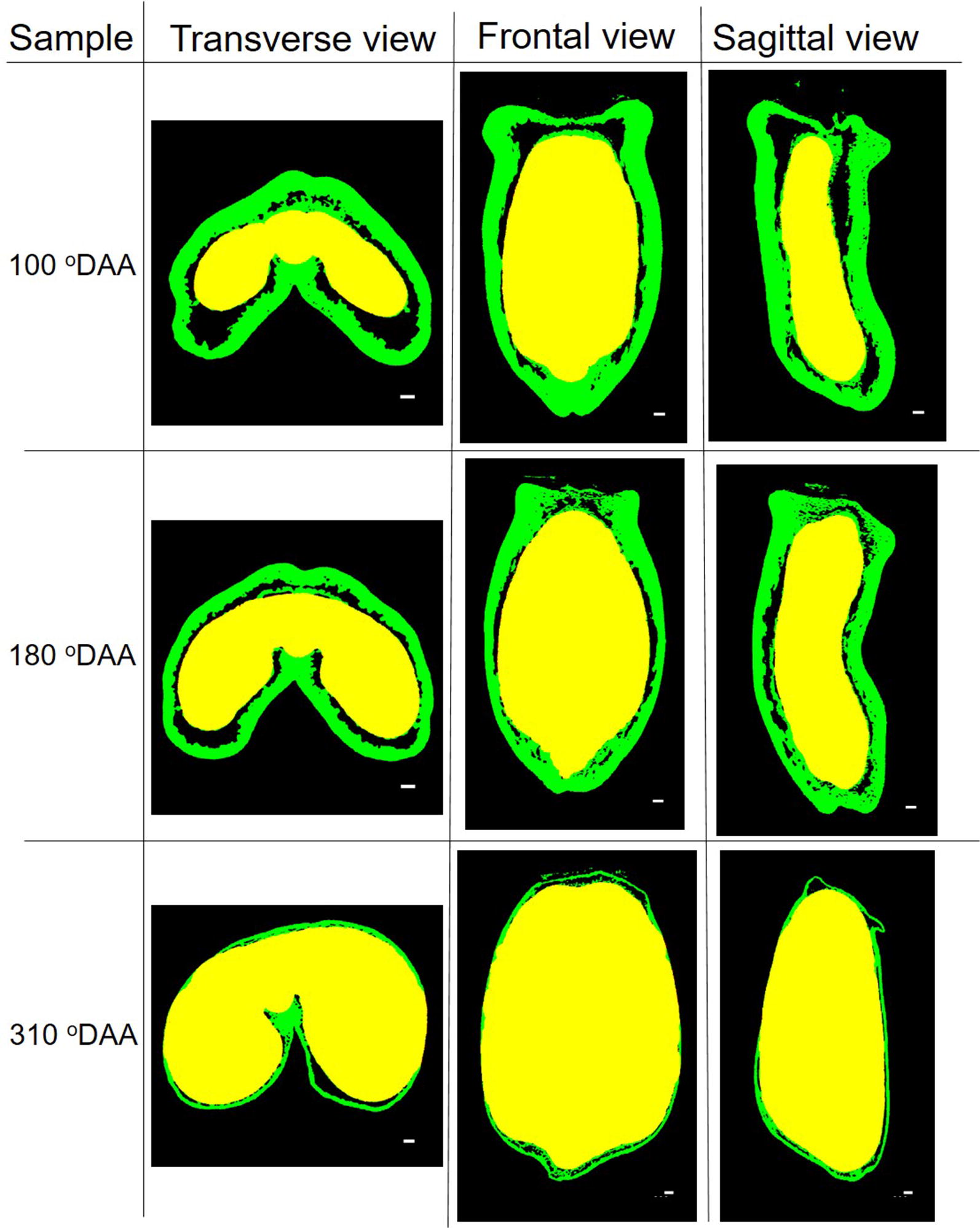
Results of wheat outer and inner tissues segmentation in several developmental stages. The green region indicates the wheat outer tissues and the yellow region indicates the wheat inner tissues. Scale bar is 200 μm

The proposed segmentation pipeline (Fig. 2) was conducted to all μCT image data and efficiently identified grain versus non grain components. The grain outer and inner compartments in all stages were also partitioned by the method described in Fig. 3. The performance depends on the distribution of voids and the thickness of the outer compartment within the grain. In particular, it was not possible to segment correctly the inner compartment at the two first stages of development. At 100 °DAA, the region of inner tissues was covered by a thick outer compartment; dark regions surround the inner compartment except on the vascular bundle region and at the bottom of the grain as shown by the different views presented in Fig. 9. The inner compartment was well segmented. At 310 °DAA, the lack of voids between the inner compartment and the thin outer tissues in some regions of the grain complicated the segmentation; a part of the outer compartment was recognized as inner compartment. In addition, it was not possible to segment the embryo because of the lack of void and of contrast between the embryo and its surrounding tissues in the upper part of the embryo.

### Morphology of grain during development

**Fig. 10:**
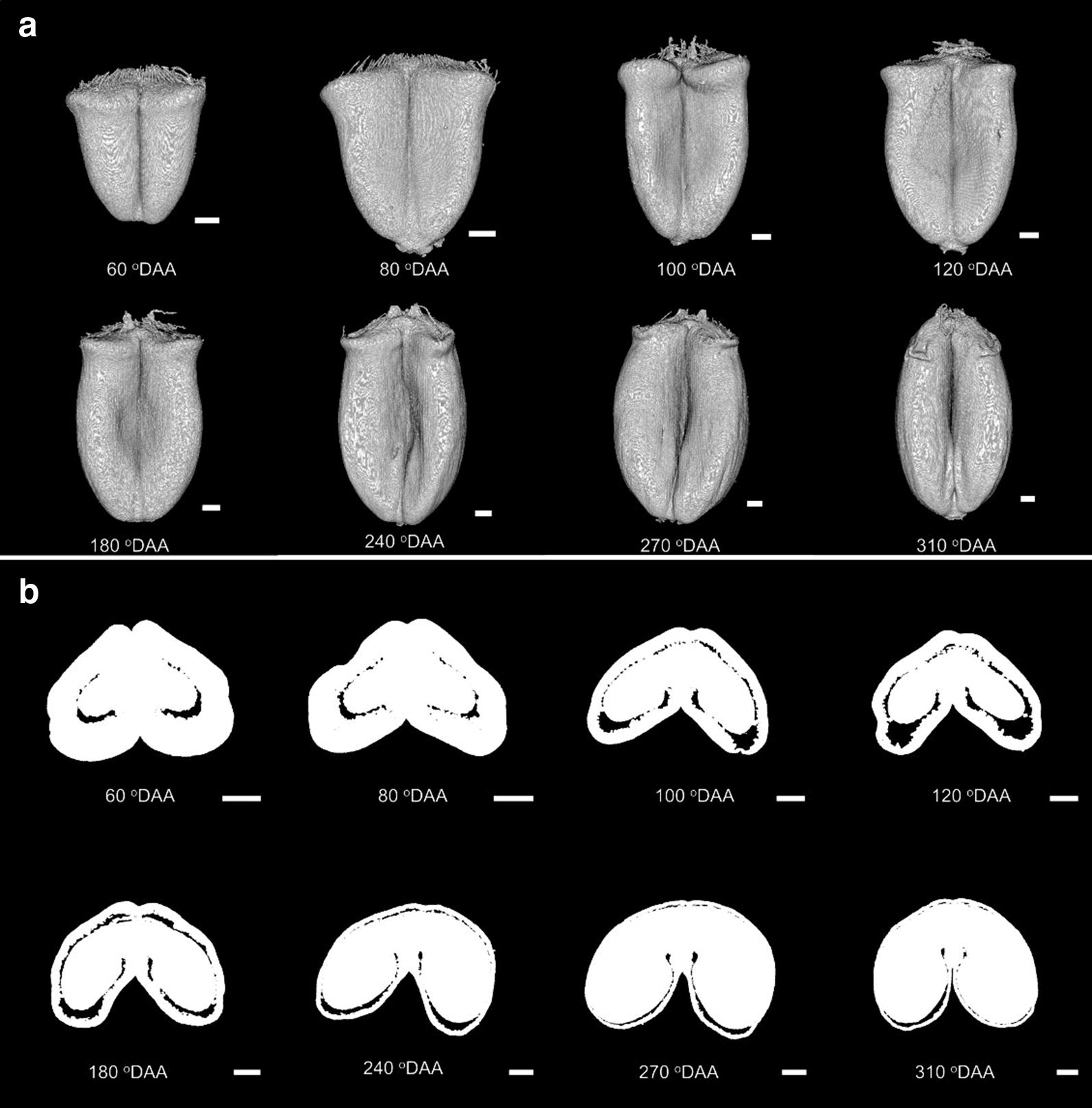
Morphology of wheat grains during development. **a** 3D views of grain. **b** Cross-section of middle part of grains. Scale bar is 600 μm

Figure 10a illustrates the 3D shape evolution of wheat grain across development. At 60 °DAA, its shape looks like an inverted triangular pyramid. From 60 to 180 °DAA, the shape of the developing grain considerably changes due to the grain elongation and enlargement of the equatorial part. From 180 °DAA, the grain shape gradually evolves to become more spherical. It presents an ellipsoidal shape at 310 °DAA.

Figure 10b illustrates the evolution of the internal structure across stages of development in the equatorial region of the grain. It provides also information on the grain shape. At 60 °DAA, the grain has a shallow crease in the ventral part of the grain but also in the dorsal part of the grain. A thick outer compartment surrounds a small region of inner tissues. In subsequent stages, the crease deepens, the inner compartment progressively fills the grain while the outer compartment at 310 °DAA represents only a thin layer. Voids occupy a small proportion of the grain at 60 °DAA, then it spreads especially at the tip of the lobes, on both sides of the vascular bundle, and in the dorsal region. The sagittal view (Fig. 9) highlights the distribution of the voids along the longitudinal axis. This view shows that large voids are present at the top of the grain early in development.

### Morphometric measures

**Fig. 11:**
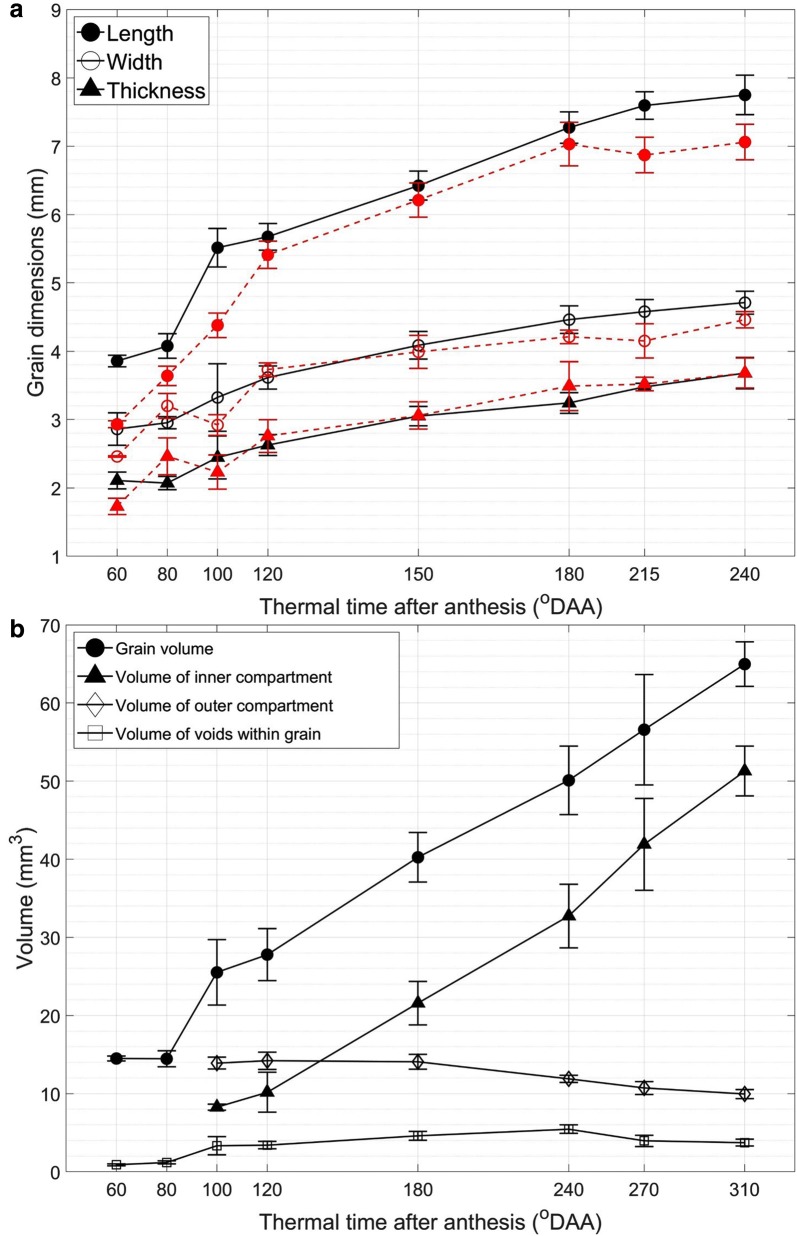
Evolution of volume and dimensions wheat grain during its development. **a** Mean grain dimensions. **b** Mean volumes of grain and its compartments. The red graph in **a** is the result measured manually using a macroscope (mean ± std)

The dimensions and volumes of wheat grain as well as its internal compartments were calculated from segmented 3D images (Additional files 3, 4). The developing wheat grains increased in length rapidly, while the width and thickness rose steadily (Fig. 11a). The grain length first increased rapidly up to 100 °DAA, especially between 80 and 100 °DAA. The elongation then still increased but more slowly and the length reached 7.8 ± 0.3 mm at 310 °DAA. Through the investigated stages of development, the grain mainly elongated with growth in the other dimensions being substantially less important. The computed values of grain dimension were remarkably close to the values obtained using a macroscope, slightly higher values for grain length were obtained for the earliest and latest stages. The computed dimensions follow classical curve reported in the literature for wheat [17, 21].

After anthesis, there was also significant rise in volume of the whole grain (Fig. 11b). The sudden increase in grain volume was observed from 14.5 ± 0.32 mm^3^ at 60 °DAA to 65 ± 2.9 mm^3^ at 310 °DAA. The grain volume almost doubled between 80 to 100 °DAA before a slower volume increase in the next 20 °DAA. After that, the evolution of the grain volume followed a linear trend up to 310 °DAA in accordance to previously published results [17, 21]. Similarly, a dramatic increase in the computed volume of inner compartment is illustrated in Fig. 11b. At 100 °DAA, it accounted for one third of the grain volume. The amount of the inner compartment kept increasing and its volume reached 51.3 ± 3.2 mm^3^ at 310 °DAA, accounting for nearly 80% of the grain volume to become the major compartment. Between 100 and 240 °DAA, the volume of inner compartment increased concomitantly with the volume of the whole grain. In contrast, the volume of the outer compartment remained stable around 14 mm^3^ until 180 °DAA and decreased afterwards. From 100 to 120 °DAA, it accounted for over 50% of grain volume, but only 15% at 310 °DAA. In addition, Fig. 11b presents the computed volume of voids within the grain. In the first stages, there was a very small proportion of voids then its volume rose and reached a peak of 5.46 ± 0.55 mm^3^ at 240 °DAA, then it decreased. It is noteworthy that from 240 °DAA, the volume of voids began to fall down while the growth of the inner compartment accelerated.

### Crease’s morphology

**Fig. 12:**
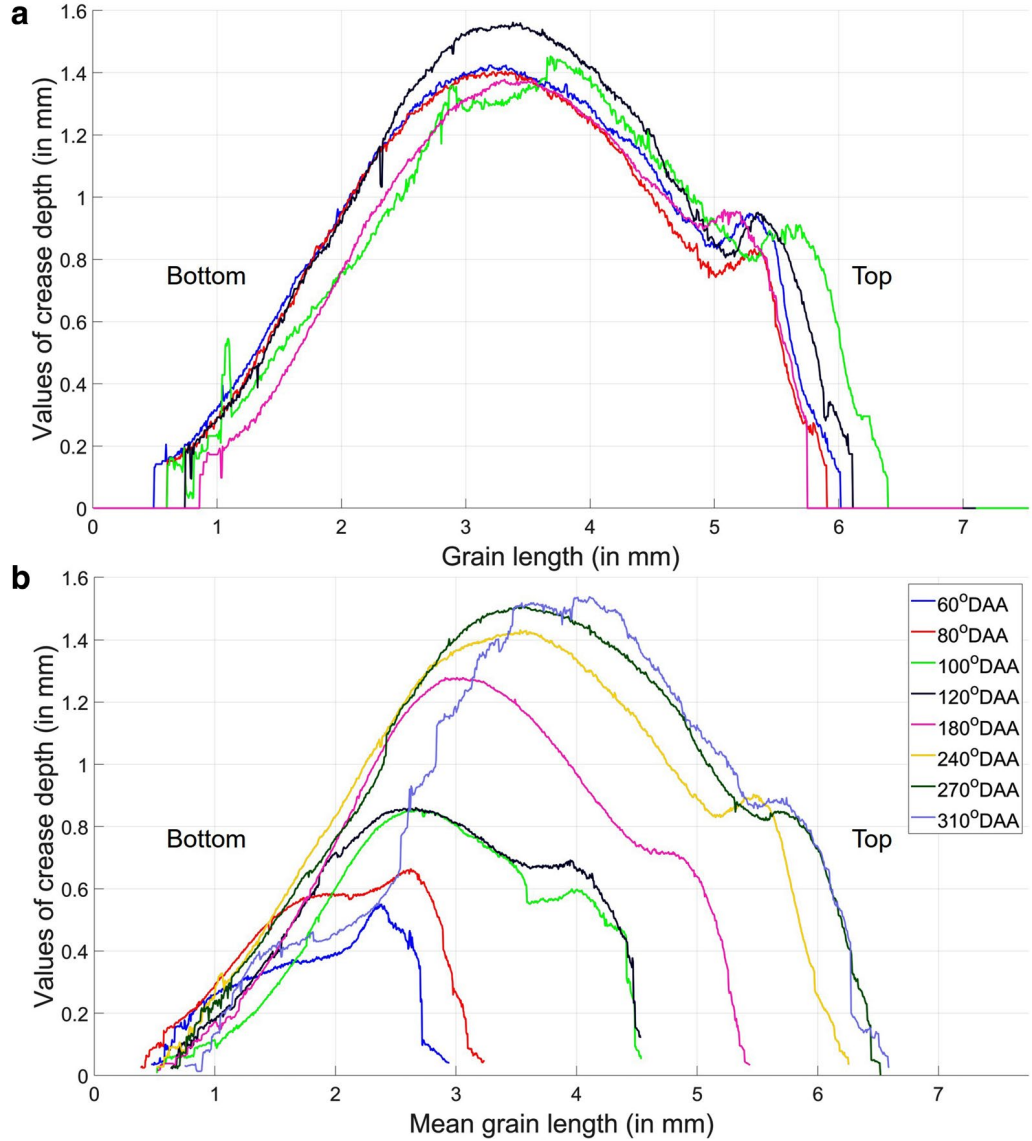
Distribution of crease depth values of all samples along the length of wheat grains. **a** Distribution of crease depth values of 5 replicates at 240 °DAA. **b** Distribution of MEAN crease depth values of all samples

The crease’s morphology was measured by calculating the distribution of crease depth values along the grain length. Figure 12a shows the variation of crease depth values of five replicates at 240 °DAA from the bottom to the top of the grains. Two bulges can be noticed in the shape of the curve of crease depth values (Fig. 12a): the more convex peak is located in the middle of the wheat grain and one less convex is located at the top of the grain.

Figure 12b illustrates the evolution of those two bulges in grains at different developmental stages. At 60 °DAA and 80 °DAA, the peak located at the top of the grain is deeper than the peak located at the middle of the grain. In contrast, the deepest point coincides approximately with the middle of the grain from 120 °DAA. The changes in the shape of the crease depth correspond to modifications observed for the grain shape as described in Fig. 12a. At 60 and 80 °DAA, the curve shape resembles a triangle while the 3D views of the same grains evoke an inverted triangular pyramid. From 120 °DAA, the distribution of crease depth values has the form of a parabola if the bulge at the top of the grain is omitted. It fits with the ellipsoid shape of the wheat grain. In addition, Fig. 12b shows that the curve of crease depth values at 310 °DAA fluctuated from the bottom to the middle of the wheat grain. This fluctuation indicates errors in the crease detection as illustrated in Additional file 5. The errors are caused by the narrow, deep crease at this stage with the two lobes of the wheat grain touching each other (see the bottom of the 3D wheat grain at 310 °DAA in Fig. 12a). The maximal value of the crease depth increases during development especially from 120 to 270 °DAA (Fig. 12b).

### Applicability of the proposed workflow to other datasets

In order to evaluate the applicability of the proposed image processing and analysis pipeline, we adapted our source code (Additional file 2) to the publicly available dataset of Hughes’s et al. [44]. This dataset contains tomography data corresponding to dry wheat spikes containing grains of another wheat genotype. As a proof of concept, we analyzed one spike. The result confirmed the feasibility of our methods (see in the Additional file 6).

## Discussion

In the context of global warming and with a growing human population, securing food supply is today a major challenge. Cereals are important staple food for which grain yield and quality have to be improved to meet the demand. Grain size and shape are among the grain traits targeted for breeding to improve the stability of grain yield as well as to efficiently manage stable grain processing. Grain size and shape are set during carpel and grain development. There is a need to develop methods to measure these grain traits as well as to investigate changes occurring during grain development (in external and internal structures) in optimal conditions and in response to biotic or abiotic stresses.

In this article we have presented the great utility of X-ray μCT for non-invasive visualization and quantification of morphological characteristics of the wheat grain and its internal structures across development under optimal conditions.

Acquisitions of wheat grain images by μCT provided images of high quality with minimum sample preparation. The spatial resolution varying between 4 and 15 μm/pixel enables to visually identify inner and outer tissue compartments, as well as the embryo (Fig. 8). The outer compartment is separated from the inner compartment by discontinuous voids. The minimum sample preparation reduces the risk of grain deformation or reconstruction artifacts, thus providing an accurate representation of the shape of the grain and of its compartments. The different cell layers that can be recognized on microscopy images are however hard to distinguish on μCT images (Fig. 7). Moreover, 3D images were generated quickly and accurately with minimum sample preparation. The acquisition took around 30 minutes for a single grain with a resolution high enough to discriminate grain compartments. Thus, it is considered as much faster than 2D imaging techniques such as light microscopy and confocal microscopy [24–26] in processes of 3D reconstruction.

The proposed segmentation method based on morphological opening allowed for automated segmentation of the grain and of the inner and outer tissue regions. The segmented images were visually found to correspond to the expected regions. However, some limitations were encountered. It was not possible to completely extract the inner compartment when its thickness was smaller than that of the outer compartment. It was the case for the two first stages (60 and 80 °DAA) and at the very top as well as at the bottom of the grains. The boundary between outer and inner compartment in the region of the vascular bundle was also not favorable since the segmentation tool determined a boundary constrained by its spherical form and its diameter. The discontinuity of voids made other segmentation techniques based on voxel intensity such as active contour inefficient. In addition, with our current resolution setup, it was impossible to segment several tissues such as the embryo which is therefore included in the inner compartment (Fig. 8).

Imaging acquisition at much higher resolutions is possible, by taking advantage of synchrotron facilities. A better spatial resolution as well as signal over background ratio can be obtained, allowing more contrast between tissues [27], but there are drawbacks such as limited access to the facility, reduced field of view and enormous data files difficult to manage. Another possibility is to merge information of several imaging modalities. For example, microscopy provides rich information about cell and tissue morphology of specific layers, but is limited to 2D images. A multiscale approach could be considered, where 3D μCT images would characterize the spatial distribution of the different tissues, and microscopy images would add details about specific regions [51, 52].

The X-ray μCT imaging technique enabled us to obtain accurate 3D images and relevant information about morphometrics of individual grain without physiological dissection. By using appropriate software, the 3D images enable us to observe the grain internal structures easily in all directions and all positions. The 3D rendering also helps analyze grain shape. Here it clearly highlighted an evolution of the grain shape during development from a triangular form to an ellipsoid. However this is a first step since statistical shape modeling should provide a better description of these changes.

The morphometric features such as grain dimensions and volumes extracted from 3D images were similar to that obtained from manual measurements, here macroscope imaging, although the computed grain length was slightly higher (Fig. 11). It could be explained by the presence of the brush on the top of wheat grains which makes accurate measurement of grain length difficult by using a macroscope. The great similarity between manual and automated measurements (especially for width and thickness), made before and after μCT acquisition respectively, make any major tissue dehydration during X-Ray μCT very unlikely. Thus this method is reliable to get accurate whole grain dimensions. The computed values follow the classical curves described in the literature [21].

From μCT images of the grains, new information was revealed. The volume of the internal compartment was measured for the first time. The values must be considered as approximations since the automatic 3D segmentation discriminated the outer and inner compartments for the major part of the grain but not at the extremities and in the vascular bundle region. The volume of the inner compartment (mainly the endosperm) evolves across development and it follows the volume of the whole grain. The presence of void (empty space) within the grain was evidenced from the first investigated stages of development. Voids in the mesocarp results from the lysis of this tissue during development. It was suggested that this programmed cell death could provide space for the growing internal compartment and enzymes involved were recently identified in barley [53]. Barley lines down-regulated for one of this enzyme exhibited smaller grains at maturity. Our method allowed evaluating the volume of void within the wheat grain and showed that this volume increased up to 240 °DAA, then it decreased while the growth of the volume of the inner compartment seemed to accelerate. These findings are in agreement with the role of voids being space made to allow the expansion of the internal compartment. μCT images also revealed that the endosperm cavity is entirely filled since no dark regions are evidenced in contrast to what is usually observed on microscopy images. This discrepancy is certainly due to the sample preparation procedure that includes cuttings and washings steps leading to tissue distortion and loss of physiological liquids. Studies on wheat and barley showed that the endosperm cavity contains sucrose and fructans [22, 54].

New information on crease morphology was also obtained by providing a basic description of its depth along the length of the grain and of its variations observed during development. In the literature, image analysis of the crease was conducted for crease detection to compare the crease depth and morphology of two wheat lines [43] and to reveal disease such as black spots that infect particularly the crease [55]. The crease depth was one of the parameter calculated by Mabille and Abecassis to elaborate a model of mature wheat grain morphology with respect to milling yield [56]. Nevertheless, there are also still other significant morphometries of the wheat grains that cannot be measured currently such as the openness of the crease; changes in size and shape of specific regions, e.g. the lobes; and the thickness of the outer layers along the grain (the thickness can be measured in cross sections). Development on specific 3D image processing techniques could be performed to overcome these limitations.

In this study we have analyzed the morphology of the developing wheat grain grown in optimal conditions in a controlled green-house. The experiment was conducted on several replicates at the same stages of development harvested on different wheat spikes but at equivalent position in the spikelet and in the spike to limit variability but variation of morphology was observed among replicates. The method developed here could contribute to evaluate the variability in the morphology of the developing grain, within a spike between different cultivars, in old wheat lines (landrace) or, in response to environmental variations (such as heat or biotic stress). For instance, heat stresses during seed development affects seed weight in many crops such as wheat [57]. The proposed method could help to evaluate the effects of a heat stress on the growth and filling of the inner compartment composed mainly of the endosperm in different genotypes, and help identify stress resilient genotypes.

## Conclusion

We demonstrate applicability of the non-destructive, non-invasive X-ray μCT imaging technique in capturing structural information of wheat grain during development. We then developed image processing methodologies to study the morphology of developing wheat grains. A new method to measure crease’s morphology is also defined to describe the wheat grain. The results of quantitative analyses reveal remarkable developmental and morphological information of wheat grain in periods of growth. This quantitative description of morphology will enable to create 4D (three-dimension + time dimension) information about anatomy and morphology of wheat grain growth and shape during its development.

## Additional files

**Additional file 1.** Overview of dataset used.

**Additional file 2.** Image processing and analysis workflow. This file contains all image processing and analysis source code and adetailed instruction explaining input and output of our method.

**Additional file 3.** File contains statistical result of grain dimensions.

**Additional file 4.** Statistics of volumes of whole grains and internal compartments.

**Additional file 5.** Figure shows crease detection on several μCT cross-sectional slices.

**Additional file 6.** Demonstration of the applicability of the proposed image processing and analysis workflow on a publicly availabledataset.

## Data Availability

The datasets used and/or analysed during the current study are available from the corresponding author on reasonable request.

## Abbreviations

μCT: micro computed tomography
4D: four-dimensional
3D: three-dimensional
2D: two-dimensional
mm: millimeter
°DAA: degrees-days after anthesis
cv: cultivar
