# Supplementary figures and images for: Use of X-ray micro computed tomography imaging to analyze the morphology of wheat grain through its development
Source: Plant Methods. 2019 Jul 31;15:84. doi: 10.1186/s13007-019-0468-y (PMC6668075; doi:10.1186/s13007-019-0468-y)

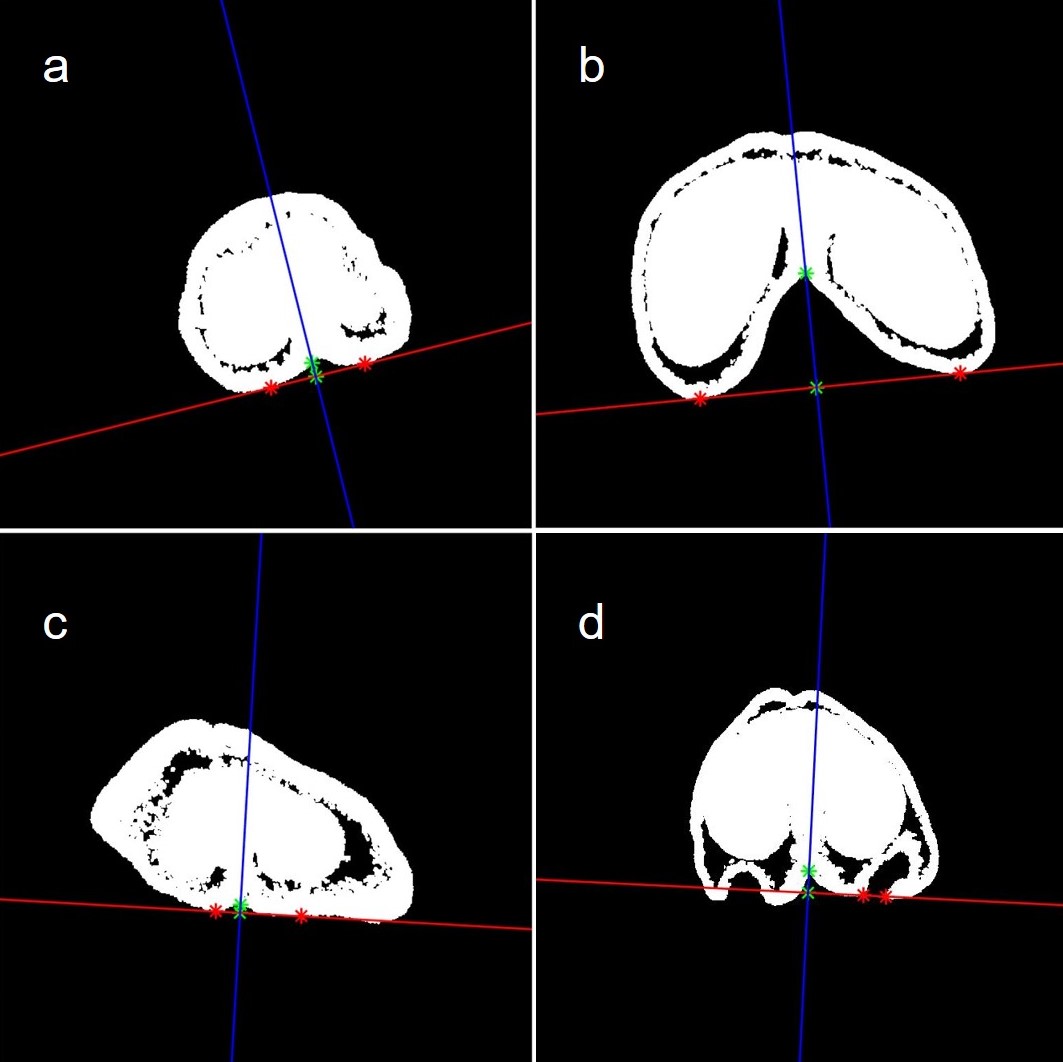

Supplement: Supplementary file 5 — Additional file 5. Figure shows crease detection on several μCT cross-sectional slices. [file 13007_2019_468_MOESM5_ESM.jpg]
