# Supplementary material for: Use of X-ray micro computed tomography imaging to analyze the morphology of wheat grain through its development
Source: Plant Methods. 2019 Jul 31;15:84. doi: 10.1186/s13007-019-0468-y (PMC6668075; doi:10.1186/s13007-019-0468-y)
Supplement: Supplementary file 6 — Additional file 6. Demonstration of the applicability of the proposed image processing and analysis workflow on a publicly availabledataset. [file 13007_2019_468_MOESM6_ESM.pdf]

Demonstration of utility of proposed μCT image processing and analysis workflow

Thang Duong Quoc Le

thang-duong-quoc.le@inra.fr

INRA UR1268 Biopolymers Interactions Assembly

May 24, 2019

Contents

1 Introduction **2**

2 Extraction of 3D wheat grain **2**

3 Extraction of grain dimensions **4**

4 Estimation of distribution of crease depth along grain length **6**

5 Conclusions **8**

References **9**

1 Introduction

This report demonstrates the utility of our proposed μCT image processing workflow for a particular public dataset [1].

There are major differences in the μCT image acquisitions between Hughes et al. [1] and ours (Table 1)

No.	Hughes et al.'s μCT image acquisition	Our μCT image acquisition
1	Images of mature grains	Images of grain in early stages of development from 60 to 310°DAA
2	Each image file is a wheat spike	Each image file is a single wheat grain
3	The resolution is 68.8 μm/pixel	The resolution varies between 4.4 and 15 μm/pixel

Table 1: Differences in μCT image acquisition

The differences in μCT image acquisition (Tab. 1) lead to differences between image processing methodologies. Therefore, we validate the source code of our proposed μCT image processing and analysis workflow for our own dataset to the public dataset by following steps:

1. Randomly choose an image file from the dataset (Tab. 2).
2. Extraction of 3D wheat grain.
3. Extraction of morphometric features: length, width, thickness and distribution of crease depth along the length of grain.

Name	Segment	Foldername	Filename	Size
01178	top	1244	1237	1018
01178	bottom	1245	1238	1171

Table 2: A μCT image of a wheat spike used for the validation.

2 Extraction of 3D wheat grain

The key difference between two image processing methods is choosing an appropriate threshold value that is able to filter the grain data from non-grain data and noise in each slice of μCT data.

Figure 1 illustrates results of our segmentation on the μCT raw image and comparing with the ground truth (Fig. b) available in the folder `Post_processed_tifs` of the public dataset [1]. Looking at the blue outline of segmented grains (Fig. c), it seems to be over segmented a little

bit because the threshold value from our method may be a little smaller than pixel intensity of individual grains. However, compare with the ground truth, the individual grains are well extracted from the raw image and their over shapes are maintained (Fig. d).

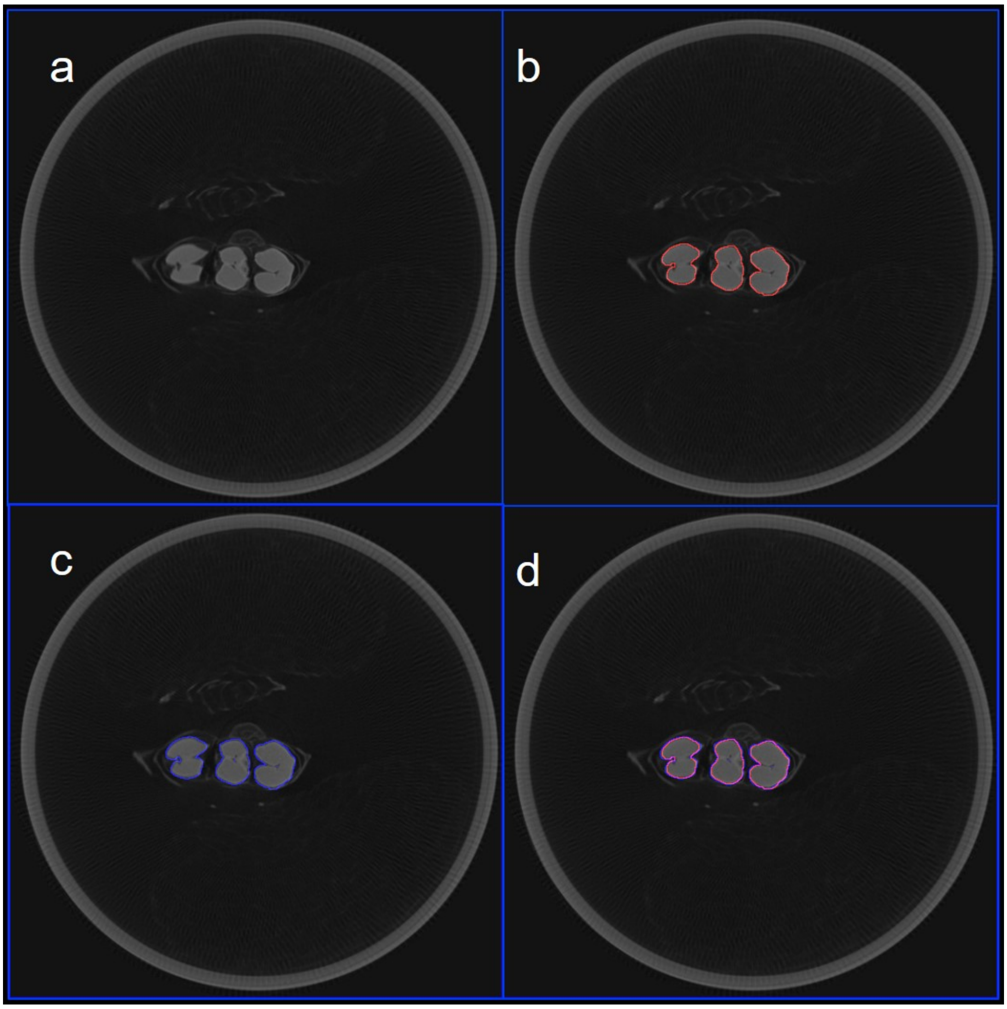

Figure 1: Wheat grain segmentation. **a** The raw μCT image; **b** Superimposition of the segmented μCT image from ground truth in [1] over a; **c** Superimposition of the segmented μCT image from our method over a; **d** Superimposition of b and c over a

To estimate the accuracy of our method, grain number in the wheat spike is taken into account. The number of grains we detected exactly the same as the number of grains provided in the ground truth data (available in folders `Data_files\00001244` and `00001245`) (Tab. 3). Meanwhile, Hughes counts lesser than 2 individual grains

Treatment No.	Segment	Hughes's ground truth	Hughes's method	Our methods
01178	top	28	26	28
01178	bottom	36	36	36

Table 3: Number of grains on wheat spike by our methods comparing with Hughes's data

.

3 Extraction of grain dimensions

3D images of wheat grains were acquired with various positions and orientations because of their positions on wheat spike (Fig. 2a). Then, it is difficult to measure morphometric features of individual wheat grains. Therefore, the 3D images of binary grains were aligned into a predefined position (Fig. 2b).

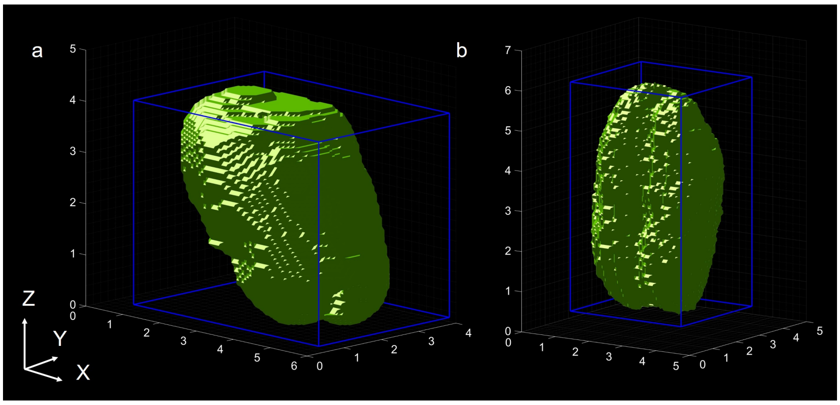

Figure 2: Alignment of an individual grain. **a** The grain before alignment; **b** The grain after alignment. (mm)

According to the available source code of Hughes et al., the length of an individual grain is the total number of slices containing the grain, while grain width and thickness were estimated by taking a cross-section of each grain and measuring major and minor 2D axis of an fitted ellipse respectively (Fig. 3 a). In contrast, in our approach, grain length was computed as the length of the bounding box fitted on the aligned wheat grain. In order to estimate the width and

thickness, 2D bounding boxes were fitted on the group of μCT cross-sections around the middle of grain. Then, the width and thickness were computed as the average dimensions of those 2D bounding boxes (Fig. 3 b).

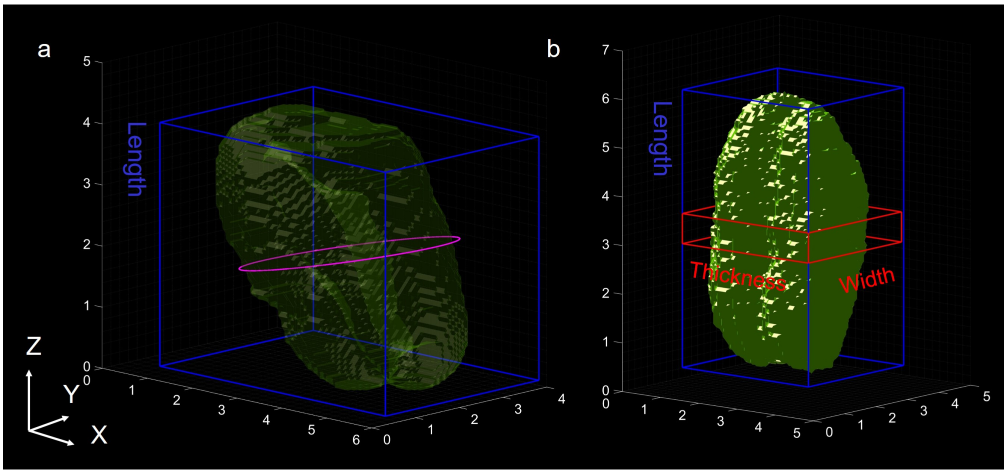

Figure 3: Morphometric measures of wheat grain. **a** The measurement of grain dimensions in [1]; **b** The measurement of grain dimensions in our method. (mm)

The differences in the estimation of grain dimensions between two methods cause the differences in values of grain dimensions (Tab. 4). Although we obtain the larger values of grain length, the values of grain width and thickness are very close to the values obtained by Hughes's method. In particular, we obtain the values of grain dimensions which have less variability than values from Hughes's method because of the alignment step.

Grain No.	Hughes's method			Our method		
	Length	Width	Thickness	Length	Width	Thickness
1	4.82	3.06	2.54	4.88	3.04	2.57
2	4.33	2.52	2.26	5.50	2.61	2.45
3	4.75	3.30	3.08	5.09	3.28	3.33
4	4.13	2.78	2.27	5.50	2.64	2.25
5	4.40	2.36	2.08	4.88	2.58	2.24
6	5.09	3.61	2.74	5.50	3.36	2.81
7	3.51	2.90	1.99	5.16	2.57	2.28
8	5.37	3.35	2.62	5.50	3.18	2.84
9	4.95	3.43	3.13	5.64	3.47	3.28
10	4.06	3.84	3.16	5.71	3.25	2.84
Mean	4.54	3.12	2.59	5.34	3.00	2.69
STD	0.56	0.48	0.44	**0.31**	**0.36**	**0.40**

Table 4: Measurement of dimensions of several grains located on a same spike. (mm)

4 Estimation of distribution of crease depth along grain length

The figure 4 shows the distribution of crease depth along the length of several individual grains. Although it works (Fig. 5a, b), there are also errors in crease detection (Fig. 5c, d). Most of the errors come from false identification of region that contains crease position.

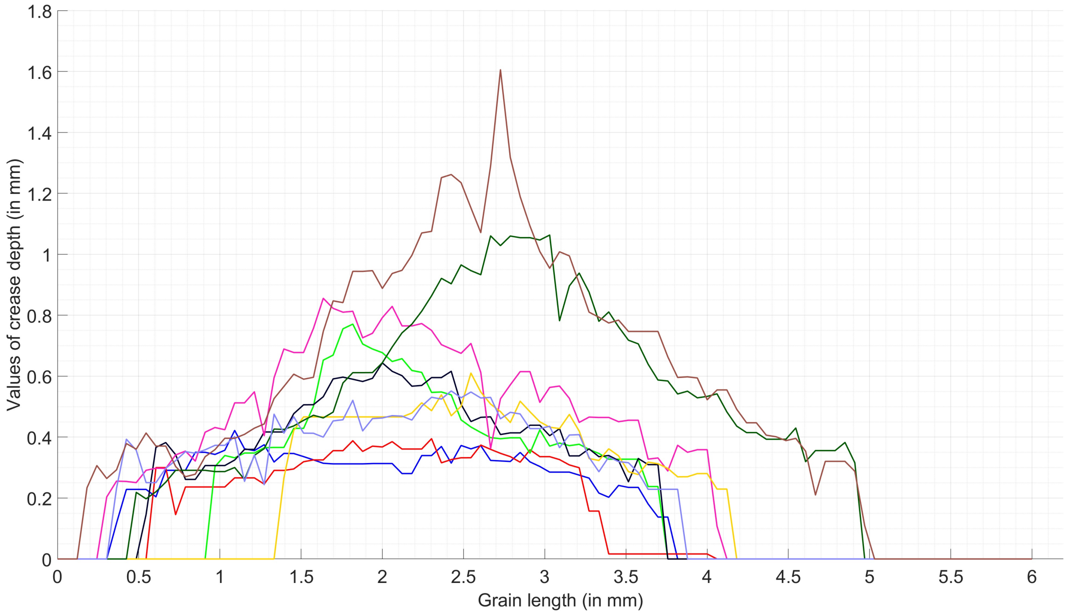

Figure 4: An example of the distribution of crease depth along the length of several individual grains in the same wheat spike. (mm)

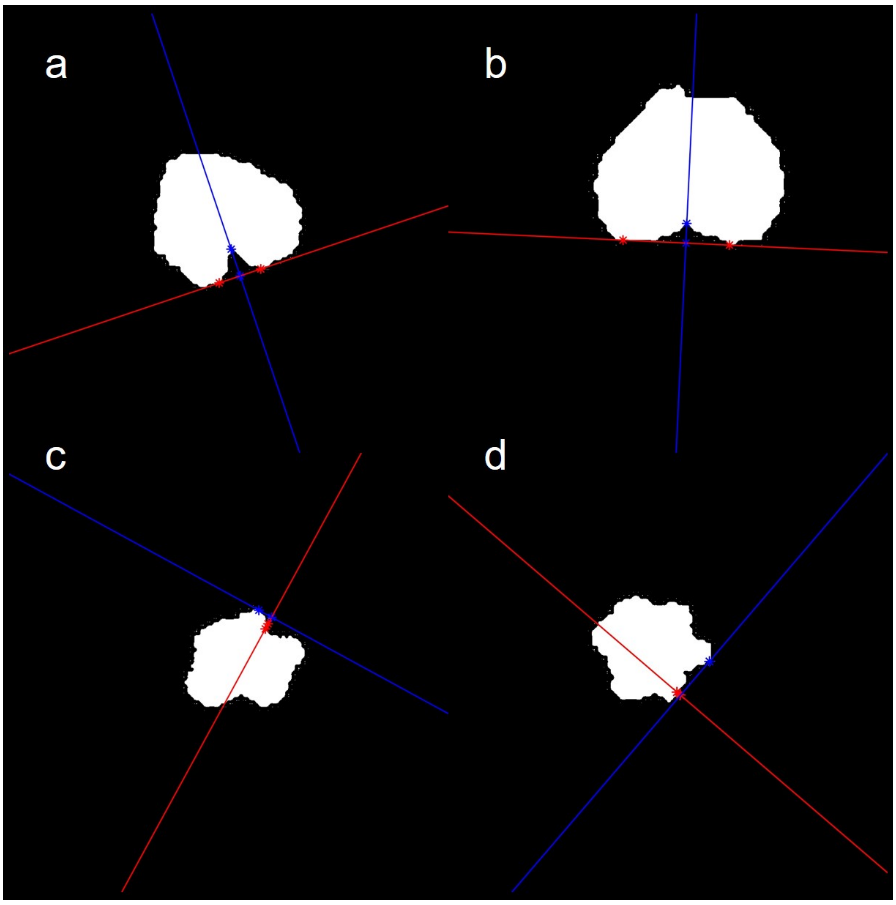

Figure 5: Crease detection on several μCT cross-sectional slices.

5 Conclusions

In this report we adapted our μCT image processing workflow to identify wheat grains and measure grain's morphometric features such as length, width and thickness on a public dataset. Our method is able to exactly extract the 3D individual grains from a μCT image of a wheat spike. The result shows that our method is able to count grains as accurately as ground truth of the dataset. The quantitative results on morphometries of grains show the applicability of our method in obtaining values of grain dimensions with smaller deviation compared with Hughes's methods.

The experimental result on computation of crease depth also shows that our method is feasible on another dataset. However, it requires further improvement for identifying the region that

contains the crease position in mature grain. In addition, the mature grain has very narrow, deep crease with the two lobes of the wheat grain touching each other (Fig. 1). It causes the errors on crease detection and computation of crease depth.

References

[1] Hughes N, Askew K, Scotson CP, Williams K, Sauze C, Corke F, et al. Non-destructive, high-content analysis of wheat grain traits using X-ray micro computed tomography. Plant Methods. 2017 Nov;13(1):76.
